# The Need for Guidelines in Asplenic Patients Undergoing Total Joint Arthroplasty: A Case Report

**DOI:** 10.1155/2012/147042

**Published:** 2012-03-26

**Authors:** S. R. Shaarani, D. Collins, J. M. O'Byrne

**Affiliations:** ^1^Department of Orthopaedic Surgery, Sports Surgery Clinic, Santry Demesne, Dublin 9, Ireland; ^2^Department of Orthopaedic Surgery, Cappagh National Orthopaedic Hospital, Finglas, Dublin 11, Ireland

## Abstract

There are currently no guidelines for splenectomy patient undergoing total joint arthroplasty. We present a case history of a 63-year-old man with a history of splenectomy that underwent a total knee arthroplasty with standard intravenous antibiotic prophylaxis. Two weeks postoperatively, he developed a prosthetic joint infection and followed the standard antimicrobial management with intravenous and oral antibiotics prior to having revision surgery. We propose that there are guidelines to properly manage these patients in the pre- and perioperative setting following an orthopaedic procedure.

## 1. Introduction

Total joint arthroplasty is currently a procedure on the rise in Europe. The rate of the number of surgeries performed on the continent from 1998 until 2008 per 100,000 population has increased from 127 to 168 in total hip arthroplasties (THA) and from 57 to 157 in total knee arthroplasties (TKA). This is a rise by approximately 16% in THA and 50% in TKA over the ten-year period [1]. The presence of medical comorbidities such as coronary artery disease, pulmonary disease, obesity, and diabetes has increased the risk of perioperative and late complications [2]. This is further confounded with additional risks of immunocompromised patients and those susceptible to systemic infection [3]. 

The British Association for Haematology has updated their guidelines in 2011 for the prevention and management of infection in asplenic and hyposplenic patients [4]. Patients with anatomical or functional asplenia have a significantly increased lifelong risk for infections involving encapsulated bacteria such as *Streptococcus pneumonia* and *Haemophilus influenzae* type [5]. The current evidence supports patient education, up-to-date vaccinations, and the continued use of penicillin prophylaxis up to the age of 16 years old and those over 50 years old. However, the consensus for prophylactic antibiotics and preventive measures remains a void in most surgical settings including dental procedures. In the orthopaedic surgical setting, there is a greater need for guidelines in the pre- and perioperative management of asplenic patients.

We present a case of a 63-year-old male with a history of splenectomy secondary to trauma and type 2 diabetes mellitus who developed a prosthetic joint infection (PJI) two weeks after the primary TKA. To our knowledge, this is the first reported case of periprosthetic joint infection in a patient with both a history of splenectomy and type II diabetes.

## 2. Case History

 A 63-year-old male presented to the outpatient with ongoing persistent pain in his left knee. A knee arthroscopy was performed which showed grade IV changes in femoral condyles and medial tibial plateau, as well as degenerative changes in the medial meniscus. This was debrided, but the patient opted for a total knee replacement the following year.

 Patient was preassessed prior to orthopaedic elective surgery and had several comorbidities: (i) posttraumatic splenectomy, on erythromycin 250 mg daily for prophylaxis and up-to-date vaccinations, (ii) type II diabetes mellitus for >20 years with HbA1c 6.6 and fasting glucose 10.9 mmol, and (iii) ischaemic heart disease with cardiac stents patent in recent angiogram and ankle-brachial index at normal values. Preoperative methicillin-resistant staphylococcus aureus (MRSA) swab test was clear of infection. The patient received standard prophylactic dose of intravenous 2nd-generation cephalosporin before his procedure.

Following overnight fast, preoperative blood glucose level was 7.2, and a glucose-potassium-insulin (GKI) infusion was commenced at 6 am. The operation was a routine knee arthroplasty for varus gonarthritis but required a TAG anchor in medial distal attachment of the patella tendon. On day 4 after TKA, patient had pyrexia of 37.7 degrees Celsius which was only documented on one episode during the day. White cell count was elevated from 8.3 preoperatively to 19.7 on day 4 postoperatively. Patient had no further episodes of pyrexia as an inpatient and, hence, discharged.

Two weeks postoperatively, the patient was diagnosed with a superficial surgical site infection and was treated by two surgical debridement procedures, application of a vacuum-assisted compression (VAC) dressing and intravenous antibiotics. MRSA swab test was positive in the surgical wound, nasal and groin sample, and this was appropriately treated locally and systemically. Patient was commenced on intravenous vancomycin; however, oral rifampicin was put on hold after initiated as patient developed an allergic reaction. Despite one month of broad-spectrum antibiotics, the decision was made by the orthopaedic surgeon to replace the polyethylene insert of the TKA due to breakdown of the surgical wound. Hence, a fasciocutaneous flap was performed by a plastic surgeon to cover the surgical wound dehiscence within the same procedure. Patient was discharged from hospital with oral linezolid. The wound required a further debridement and split-skin graft 2 months later.

**Figure 1:**
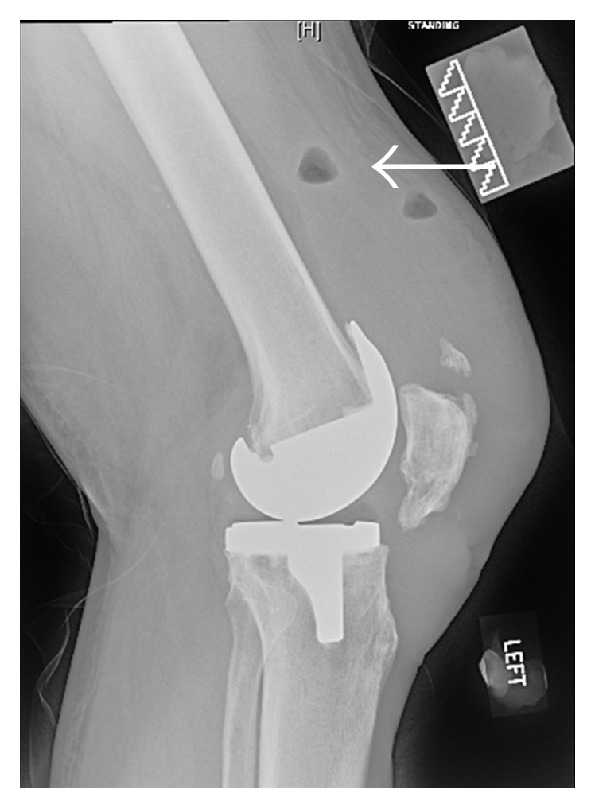
Lateral left knee X-ray showing a patella baja, fluid collection near the quadriceps tendon, and the presence of gas in the thigh, with query infection.

**Figure 2:**
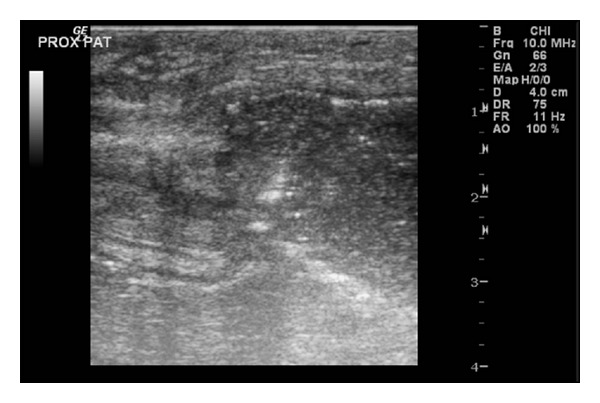
Ultrasound of the left knee showing disruption of the quadriceps tendon and fluid collection.

**Figure 3:**
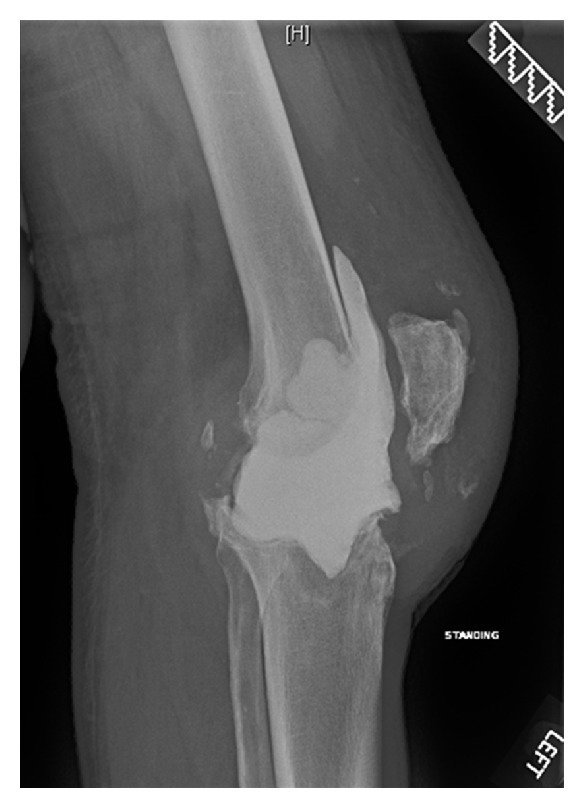
Lateral X-ray of the left knee after the first-stage revision with an antibiotic-impregnated cement spacer 6 months after initial TKA.

Three months later, there was gross swelling in his left knee and inability to perform the straight-leg test. An initial diagnosis of quadriceps tendon rupture was made clinically. X-ray and ultrasound confirmed the diagnosis but commented on the possibility of infection (Figures 1 and 2). Intraoperative findings showed copious pus material and necrotic tissue which required debridement and irrigation. The quadriceps tendon is loosely sutured back together to prevent further retraction of the tendon. Microbiologic studies revealed heavy growth of methicillin-sensitive staphylococcus aureus. MRSA was not isolated. Patient had a 1st-stage revision of the left total knee replacement after 2 weeks of intravenous antibiotics (Figure 3). He was then discharged on long-term oral flucloxacillin. He proceeded to a knee fusion with an Ilizarov external fixator. 

## 3. Discussion

Asplenic patients are at increased risk of encapsulated bacterial infections; however, they do not mount normal physiological responses such as pyrexia [6]. The effectiveness and timing of the vaccination prior to surgical procedures including orthopaedic interventions remain unknown [7]. A multidisciplinary approach which includes a haematologist and microbiologist in the preoperative setting could prevent and enhance detection of infection. The initial single episode of pyrexia and subsequent increase in white cell count should have prompted further investigations with initial septic screen, knee aspiration, and possibly a leucocyte scan. Though these are normal physiologic responses postoperatively, a low threshold of suspicion in asplenic patients may improve the detection of early stages of an infection.

The presence of diabetes mellitus in this patient significantly increases the relative risk of joint infection by 1.5–3 and requiring revision arthroplasty surgery [8, 9]. A preoperative blood glucose of less than 6.1 mmol/L (<110 mg/dL), 6.1–6.9 mmol/L (110–125 mg/dL), and greater or equal to 7.0 mmol/L (≥126 mg/dL) correlated to an incidence of periprosthetic infection of 0.44%, 0.93%, and 2.42%, respectively [10]. Even a day 1 postoperative blood glucose of 8.55 ± 1.72 mmol/L (154 ± 37 mg/dL) had a significant risk of early to late infection [11].

 The subsequent growth of MRSA is of concern as there is an increased risk for infection by encapsulated bacteria and further deterioration to severe sepsis [12]. The patient initially had negative preassessment MRSA swabs, but this was positive 2 weeks after the initial TKR. Genotyping of the MRSA infection could have been useful at this stage to ascertain whether it is a hospital or community-acquired infection. Parenteral antimicrobials are the mainstay of treatment after infection [13], but preloaded antibiotic cement [14] or direct intra-articular vancomycin could have been used to prevent the initial and progression of the prosthetic joint infection [15].

## 4. Conclusion

 This paper is of particular importance to all orthopaedic surgeons involved in elective orthopaedic arthroplasty. It highlights the importance of a multidisciplinary approach when planning for surgery in the splenectomy patient with additional comorbidities. There should be a low tolerance for investigations for any evidence of infection, as these subgroups, of patients will not have the normal physiologic response to microbial assault. Most importantly, there is a void in guidelines that need to be addressed in patients undergoing orthopaedic surgeries in asplenic patients. Future research and audit should incorporate a safe surgical pathway for this cohort of patients.

## References

[1] Health at a Glance: Europe http://www.dx.doi.org/10.1787/888932337167.

[2] Memtsoudis SG, González Della Valle A, Besculides MC, Gaber L, Sculco TP (2008). In-hospital complications and mortality of unilateral, bilateral, and revision TKA: based on an estimate of 4,159,661 discharges. *Clinical Orthopaedics and Related Research*.

[3] Powell DL, Whitener CJ, Dye CE, Ballard JO, Shaffer ML, Eyster ME (2005). Knee and hip arthroplasty infection rates in persons with haemophilia: a 27 year single center experience during the HIV epidemic. *Haemophilia*.

[4] Davies JM, Lewis MP, Wimperis J, Rafi I, Ladhani S, Bolton-Maggs PH (2011). Review of guidelines for the prevention and treatment of infection in patients with an absent or dysfunctional spleen: prepared on behalf of the British committee for standards in haematology by a working party of the haemato-oncology task force. *British Journal of Haematology*.

[5] Melles DC, de Marie S (2004). Prevention of infections in hyposplenic and asplenic patients: an update. *Netherlands Journal of Medicine*.

[6] Pizzo PA (1999). Fever in immunocompromised patients. *The New England Journal of Medicine*.

[7] Löbermann M, Boršo D, Hilgendorf I, Fritzsche C, Zettl UK, Reisinger EC (2012). Immunization in the adult immunocompromised host. *Autoimmunity Reviews*.

[8] Pedersen AB, Mehnert F, Johnsen SP, Sørensen HT (2010). Risk of revision of a total hip replacement in patients with diabetes mellitus: a population-based follow up study. *Journal of Bone and Joint Surgery B*.

[9] Malinzak RA, Ritter MA, Berend ME, Meding JB, Olberding EM, Davis KE (2009). Morbidly obese, diabetic, younger, and unilateral joint arthroplasty patients have elevated total joint arthroplasty infection rates. *Journal of Arthroplasty*.

[10] Jämsen E, Nevalainen P, Kalliovalkama J, Moilanen T (2010). Preoperative hyperglycemia predicts infected total knee replacement. *European Journal of Internal Medicine*.

[11] Mraovic B, Suh D, Jacovides C, Parvizi J (2011). Perioperative hyperglycemia and postoperative infection after lower limb arthroplasty. *Journal of Diabetes Science and Technology*.

[12] Roth A, Fuhrmann R, Lange M, Mollenhauer J, Straube E, Venbrocks R (2003). Overwhelming septic infection with a multi-resistant Staphylococcus aureus (MRSA) after total knee replacement. *Archives of Orthopaedic and Trauma Surgery*.

[13] Bradbury T, Fehring TK, Taunton M (2009). The fate of acute methicillin-resistant staphylococcus aureus periprosthetic knee infections treated by open debridement and retention of components. *Journal of Arthroplasty*.

[14] Nickinson RS, Board TN, Gambhir AK, Porter ML, Kay PR (2010). The microbiology of the infected knee arthroplasty. *International Orthopaedics*.

[15] Whiteside LA, Peppers M, Nayfeh TA, Roy ME (2011). Methicillin-resistant staphylococcus aureus in TKA treated with revision and direct intraarticular antibiotic infusion. *Clinical Orthopaedics and Related Research*.

